# Renormalization scheme dependence of the two-loop QCD corrections to the neutral Higgs-boson masses in the MSSM

**DOI:** 10.1140/epjc/s10052-015-3648-6

**Published:** 2015-09-15

**Authors:** S. Borowka, T. Hahn, S. Heinemeyer, G. Heinrich, W. Hollik

**Affiliations:** Institute for Physics, University of Zurich, Winterthurerstr. 190, 8057 Zurich, Switzerland; Max-Planck-Institut für Physik (Werner-Heisenberg-Institut), Föhringer Ring 6, 80805 München, Germany; Instituto de Física de Cantabria (CSIC-UC), Santander, Spain

## Abstract

Reaching a theoretical accuracy in the prediction of the lightest MSSM Higgs-boson mass, $M_h$, at the level of the current experimental precision requires the inclusion of momentum-dependent contributions at the two-loop level. Recently two groups presented the two-loop QCD momentum-dependent corrections to $M_h$ (Borowka et al., Eur Phys J C 74(8):2994, 2014; Degrassi et al., Eur Phys J C 75(2):61, 2015), using a hybrid on-shell-$\overline{\mathrm {DR}}$ scheme, with apparently different results. We show that the differences can be traced back to a different renormalization of the top-quark mass, and that the claim in Ref. Degrassi et al. (Eur Phys J C 75(2):61, 2015) of an inconsistency in Ref. Borowka et al. (Eur Phys J C 74(8):2994, 2014) is incorrect. We furthermore compare consistently the results for $M_h$ obtained with the top-quark mass renormalized on-shell and $\overline{\mathrm {DR}}$. The latter calculation has been added to the FeynHiggs package and can be used to estimate missing higher-order corrections beyond the two-loop level.

## Introduction

The particle discovered in the Higgs-boson searches by ATLAS [3] and CMS [4] at CERN shows, within experimental and theoretical uncertainties, properties compatible with the Higgs boson of the Standard Model (SM) [5–7]. It can also be interpreted as the Higgs boson of extended models, however, where the lightest Higgs boson of the Minimal Supersymmetric Standard Model (MSSM) [8–10] is a prime candidate.

The Higgs sector of the MSSM with two scalar doublets accommodates five physical Higgs bosons. In lowest order these are the light and heavy $\mathcal{CP}$-even *h* and *H*, the $\mathcal{CP}$-odd *A*, and the charged Higgs bosons $H^\pm$. At tree level, the Higgs sector can be parameterized in terms of the gauge couplings, the mass of the $\mathcal{CP}$-odd Higgs boson, $M_A$, and $\tan \beta \equiv v_2/v_1$, the ratio of the two vacuum expectation values; all other masses and mixing angles follow as predictions.

Higher-order contributions can give large corrections to the tree-level relations [11–13], and in particular to the mass of the lightest Higgs boson, $M_h$. For the MSSM^1^ with real parameters the status of higher-order corrections to the masses and mixing angles in the neutral Higgs sector is quite advanced; see Refs. [19–26] for the calculations of the full one-loop level. At the two-loop level [18, 27–44] in particular the $\mathcal{O}(\alpha _t\alpha _s)$ and $\mathcal{O}(\alpha _t^2)$ contributions ($\alpha _t\equiv h_t^2 / (4 \pi )$, $h_t$ being the top-quark Yukawa coupling) to the self-energies – evaluated in the Feynman-diagrammatic (FD) as well as in the effective potential (EP) method – as well as the $\mathcal{O}(\alpha _b\alpha _s)$, $\mathcal{O}(\alpha _t\alpha _b)$ and $\mathcal{O}(\alpha _b^2)$ contributions – evaluated in the EP approach – are known for vanishing external momenta. An evaluation of the momentum dependence at the two-loop level in a pure $\overline{\mathrm {DR}}$ calculation was presented in Ref. [45]. The latest status of the momentum-dependent two-loop corrections will be discussed below. A (nearly) full two-loop EP calculation, including even the leading three-loop corrections, has also been published [46–50, 52, 53]. Within the EP method all contributions are evaluated at zero external momentum, however, in contrast to the FD method which in principle allows for non-vanishing external momenta. Furthermore, the calculation presented in Refs. [46–50, 52, 53] is not publicly available as a computer code for Higgs-boson mass calculations. Subsequently, another leading three-loop calculation of $\mathcal{O}(\alpha _t\alpha _s^2)$, depending on the various SUSY mass hierarchies, was completed [54–56], resulting in the code H3m which adds the three-loop corrections to the FeynHiggs [14, 29, 57–60] result. Most recently, a combination of the full one-loop result, supplemented with leading and subleading two-loop corrections evaluated in the FD/EP method and a resummation of the leading and subleading logarithmic corrections from the scalar-top sector has been published [60] in the latest version of the code FeynHiggs.

The measured mass value of the observed Higgs boson is currently known to about $250 \,\, \mathrm {MeV}$ accuracy [5], reaching the level of a precision observable. At a future linear collider (ILC), the precise determination of the light Higgs-boson properties and/or heavier MSSM Higgs bosons within the kinematic reach will be possible [61]. In particular, a mass measurement of the light Higgs boson with an accuracy below $\sim 0.05 \,\, \mathrm {GeV}$ is anticipated [62].

In Ref. [59] the remaining theoretical uncertainty in the calculation of $M_h$, from unknown higher-order corrections, was estimated to be up to $3 \,\, \mathrm {GeV}$, depending on the parameter region; see also Refs. [60, 63] for updated results. As the accuracy of the $M_h$ prediction should at least match the one of the experimental result, higher-order corrections which do not dominate the size of the Higgs-boson mass values have to be included in the Higgs-boson mass predictions.

To better control the size of momentum-dependent contributions, we recently presented the calculation of the $\mathcal{O}(p^2\alpha _t\alpha _s)$ corrections to $M_h$ (the leading momentum-dependent two-loop QCD corrections). The calculation was performed in a hybrid on-shell/$\overline{\mathrm {DR}}$ scheme [1] at the two-loop level, where $M_A$ and the tadpoles are renormalized on-shell (OS), whereas the Higgs-boson fields and $\tan \beta$ are renormalized $\overline{\mathrm {DR}}$. At the one-loop level the top/stop parameters are renormalized OS.^2^ Subsequently, in Ref. [2] this calculation was repeated with a different result (also, a calculation in a pure $\overline{\mathrm {DR}}$ scheme as well as the two-loop corrections of $\mathcal{O}(\alpha \alpha _s)$ were presented). Within Ref. [2] the discrepancy between Refs. [1, 2] was explained by an inconsistency in the renormalization scheme used for the Higgs-boson field renormalization in Ref. [1].

In this paper we demonstrate that this claim is incorrect. The renormalization scheme for the Higgs-boson fields used in Ref. [1] is (up to corrections beyond the two-loop level) identical to the one employed in Ref. [2]. We clarify that the differences between the two results originates in a difference of the top-quark-mass renormalization scheme. While in Ref. [1] a full OS renormalization was used, in Ref. [2] the contributions to the top-quark self-energy of $\mathcal{O}(\varepsilon )$ (with $4 - D = 2\varepsilon$, *D* being the space-time dimension) were neglected, leading to the observed numerical differences. We also demonstrate how this difference in the treatment of the contributions from the top-quark mass can be linked to a difference in the two-loop field renormalization constant and explain why this difference should be regarded as a theoretical uncertainty at the two-loop level, which would be fixed only at three-loop order.

We further present a consistent calculation of the $\mathcal{O}(p^2\alpha _t\alpha _s)$ corrections to $M_h$ in a scheme where the top quark is renormalized $\overline{\mathrm {DR}}$, whereas the scalar tops continue to be renormalized OS. This new scheme is available from FeynHiggs version 2.11.1 on, allowing for an improved estimate of (some) unknown higher-order corrections beyond the two-loop level originating from the top/stop sector.

The paper is organized as follows. An overview of the relevant sectors and the renormalization employed in our calculation is given in Sect. 2. In Sect. 3 we compare analytically and numerically the results of Refs. [1, 2]. Results obtained using the $\overline{\mathrm {DR}}$ scheme for the top-quark mass are given in Sect. 4. Our conclusions are given in Sect. 5.

## The relevant sectors and their renormalization

### The Higgs-boson sector of the MSSM

The MSSM requires two scalar doublets, which are conventionally written in terms of their components as follows:

$$\begin{aligned} \mathcal{H}_1&= \left( \begin{array}{c}\mathcal{H}_1^0 \\ \mathcal{H}_1^- \end{array} \right) = \left( \begin{array}{c}v_1 + \frac{1}{\sqrt{2}}(\phi _1^0 - i\chi _1^0) \\ -\phi _1^- \end{array} \right) , \\ \mathcal{H}_2&= \left( \begin{array}{c}\mathcal{H}_2^+ \\ \mathcal{H}_2^0 \end{array} \right) = \left( \begin{array}{c}\phi _2^+ \\ v_2+ \frac{1}{\sqrt{2}}(\phi _2^0 + i\chi _2^0) \end{array} \right) . \end{aligned}$$

The bilinear part of the Higgs potential leads to the tree-level mass matrix for the neutral $\mathcal{CP}$-even Higgs bosons,

1 $$\begin{aligned} M_{\text {Higgs}}^{2,\text {tree}}&= \left( \begin{array}{ll}m_{\phi _1}^2&{} m_{\phi _1\phi _2}^2 \\ m_{\phi _1\phi _2}^2&{} m_{\phi _2}^2 \end{array} \right) \nonumber \\&= \left( \begin{array}{ll}M_A^2 \sin ^2 \beta + M_Z^2 \cos ^2 \beta &{}\quad -(M_A^2 + M_Z^2) \sin \beta \cos \beta \\ -(M_A^2 + M_Z^2) \sin \beta \cos \beta &{}\quad M_A^2 \cos ^2 \beta + M_Z^2 \sin ^2 \beta \end{array} \right) , \end{aligned}$$

in the $(\phi _1, \phi _2)$ basis, expressed in terms of the *Z* boson mass, $M_Z$, $M_A$, and the angle $\beta$. Diagonalization via the angle $\alpha$ yields the tree-level masses $m_{h, \mathrm{tree}}$ and $m_{H, \mathrm{tree}}$. Below we also use $M_W$, denoting the *W* boson mass and $s_\mathrm {w}$, the sine of the weak mixing angle, $s_\mathrm {w}= \sqrt{1 - c_\mathrm {w}^2} = \sqrt{1 - M_W^2/M_Z^2}$.

The higher-order-corrected $\mathcal{CP}$-even Higgs-boson masses in the MSSM are obtained from the corresponding propagators dressed by their self-energies. The calculation of these and their renormalization is performed in the $(\phi _1, \phi _2)$ basis, which has the advantage that the mixing angle $\alpha$ does not appear and expressions are in general simpler. The inverse propagator matrix in the $(\phi _1, \phi _2)$ basis is given by

2 $$\begin{aligned}&(\Delta _{\text {Higgs}})^{-1} \nonumber \\&\quad = -\text {i} \left( \begin{array}{l@{\quad }l} p^2 - m_{\phi _1}^2 + \hat{\Sigma }_{\phi _1}(p^2) &{} -m_{\phi _1\phi _2}^2 +\hat{\Sigma }_{\phi _1\phi _2}(p^2)\\ -m_{\phi _1\phi _2}^2 +\hat{\Sigma }_{\phi _1\phi _2}(p^2) &{} p^2 - m_{\phi _2}^2 + \hat{\Sigma }_{\phi _2}(p^2) \end{array} \right) , \end{aligned}$$

where $\hat{\Sigma }(p^2)$ denote the renormalized Higgs-boson self-energies, *p* being the external momentum. The renormalized self-energies can be expressed through the unrenormalized self-energies, $\Sigma (p^2)$, and counterterms involving renormalization constants $\delta m^2$ and $\delta Z$ from parameter and field renormalization. With the self-energies expanded up to two-loop order, $\hat{\Sigma }= \hat{\Sigma }^{(1)} + \hat{\Sigma }^{(2)}$, one has for the $\mathcal{CP}$-even part at the *i*-loop level ($i = 1, 2$),

3a $$\begin{aligned}&\hat{\Sigma }_{\phi _1}^{(i)}(p^2) =\, \Sigma _{\phi _1}^{(i)}(p^2) + \delta Z_{\phi _1}^{(i)}\, (p^2-m_{\phi _1}^2) - \delta m_{\phi _1}^{2(i)}, \end{aligned}$$

3b $$\begin{aligned}&\hat{\Sigma }_{\phi _1\phi _2}^{(i)}(p^2) =\, \Sigma _{\phi _1\phi _2}^{(i)}(p^2) - \delta Z_{\phi _1\phi _2}^{(i)}\, m_{\phi _1\phi _2}^2 - \delta m_{\phi _1\phi _2}^{2(i)},\end{aligned}$$

3c $$\begin{aligned}&\hat{\Sigma }_{\phi _2}^{(i)}(p^2) =\, \Sigma _{\phi _2}^{(i)}(p^2) + \delta Z_{\phi _2}^{(i)}\, (p^2-m_{\phi _2}^2) - \delta m_{\phi _2}^{2(i)}. \end{aligned}$$

At the two-loop level the expressions in Eqs. (3) do not contain contributions of the type (1-loop) $\times$ (1-loop); such terms do not appear at $\mathcal{O}(\alpha _t\alpha _s)$ and hence can be omitted in the context of this paper. For the general expressions see Ref. [18].

Beyond the one-loop level, unrenormalized self-energies contain sub-loop renormalizations. At the two-loop level, these are one-loop diagrams with counterterm insertions at the one-loop level.

### Renormalization

The following section summarizes the renormalization worked out in Ref. [1], based on Ref. [29]. The field renormalization is carried out by assigning one renormalization constant to each doublet,

4 $$\begin{aligned} \mathcal{H}_1\rightarrow (1 + \tfrac{1}{2} \delta Z_{\mathcal{H}_1} )\, \mathcal{H}_1, \quad \mathcal{H}_2\rightarrow (1 + \tfrac{1}{2} \delta Z_{\mathcal{H}_2} ) \mathcal{H}_2, \end{aligned}$$

which can be expanded to one- and two-loop order according to

5 $$\begin{aligned} \delta Z_{\mathcal{H}_1}&=\, \delta Z_{\mathcal{H}_1}^{(1)} + \delta Z_{\mathcal{H}_1}^{(2)} \, , \quad \delta Z_{\mathcal{H}_2} \, =\, \delta Z_{\mathcal{H}_2}^{(1)} + \delta Z_{\mathcal{H}_2}^{(2)}. \end{aligned}$$

The field renormalization constants appearing in (3) are then given by

6 $$\begin{aligned}&\delta Z_{\phi _1}^{(i)} = \delta Z_{\mathcal{H}_1} ^{(i)}, \quad \delta Z_{\phi _2}^{(i)} = \delta Z_{\mathcal{H}_2} ^{(i)}, \nonumber \\&\quad \delta Z_{\phi _1\phi _2}^{(i)} = \tfrac{1}{2}(\delta Z_{\mathcal{H}_1}^{(i)} + \delta Z_{\mathcal{H}_2}^{(i)} ). \end{aligned}$$

The mass counterterms $\delta m^{2 (i)}_{ab}$ in Eq. (3) are derived from the Higgs potential, including the tadpoles, by the following parameter renormalization:

7 $$\begin{aligned}&M_A^2 \rightarrow M_A^2 + \delta M_A^{2(1)}+ \delta M_A^{2(2)},\nonumber \\&T_1\rightarrow T_1+ \delta T_1^{(1)}+ \delta T_1^{(2)}, \nonumber \\&M_Z^2 \rightarrow M_Z^2 + \delta M_Z^{2(1)}+ \delta M_Z^{2(2)},\nonumber \\&T_2\rightarrow T_2+ \delta T_2^{(1)}+ \delta T_2^{(2)}, \nonumber \\&\tan \beta \rightarrow \tan \beta \left( 1 + \delta \tan \beta ^{(1)} + \delta \tan \beta ^{(2)} \right) . \end{aligned}$$

The parameters $T_1$ and $T_2$ are the terms linear in $\phi _1$ and $\phi _2$ in the Higgs potential. The renormalization of the *Z*-mass $M_Z$ does not contribute to the $\mathcal {O}(\alpha _s \alpha _t)$ corrections we are pursuing here; it is listed for completeness only.

The basic renormalization constants for parameters and fields have to be fixed by renormalization conditions according to a renormalization scheme. Here we choose the on-shell scheme for the parameters and the $\overline{\mathrm{{DR}}}$ scheme for field renormalization and give the expressions for the two-loop part. This is consistent with the renormalization scheme used at the one-loop level.

**Fig. 1:**
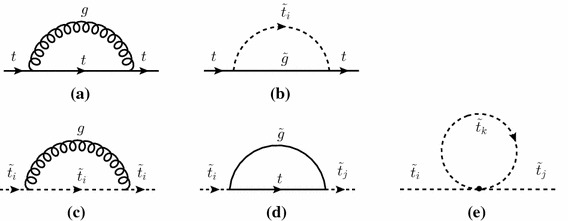
Generic one-loop diagrams for subrenormalization counterterms for the top quark (*upper row*) and for the scalar tops (*lower row*) ($i,j,k =1,2$)

The tadpole coefficients are chosen to vanish at all orders; hence their two-loop counterterms follow from

8 $$\begin{aligned} T_{1,2}^{(2)}+ \delta T_{1,2}^{(2)} = 0, \quad \text {i.e. }\quad \delta T_1^{(2)}= -{T_1^{(2)}}, \quad \delta T_2^{(2)}= -{T_2^{(2)}}, \end{aligned}$$

where $T_1^{(2)}$, $T_2^{(2)}$ are obtained from the two-loop tadpole diagrams. The two-loop renormalization constant of the *A*-boson mass reads

9 $$\begin{aligned} \delta M_A^{2(2)}= \mathop {\mathrm {Re}}\Sigma _{AA}^{(2)}(M_A^2) , \end{aligned}$$

in terms of the *A*-boson unrenormalized self-energy $\Sigma _{AA}$. The appearance of a non-zero momentum in the self-energy goes beyond the $\mathcal{O}(\alpha _t\alpha _s)$ corrections evaluated in Refs. [27–29, 35].

For the renormalization constants $\delta Z_{\mathcal{H}_1}$, $\delta Z_{\mathcal{H}_2}$, and $\delta \tan \beta$ several choices are possible; see the discussion in [67–69]. As shown there, the most convenient choice is a $\overline{\mathrm{{DR}}}$ renormalization of $\delta \tan \beta$, $\delta Z_{\mathcal{H}_1}$, and $\delta Z_{\mathcal{H}_2}$, which at the two-loop level reads

10a $$\begin{aligned}&\delta Z_{\mathcal{H}_1}^{(2)} = \delta Z_{\mathcal{H}_1}^{\overline{\mathrm {DR}}(2)} \; = \; - \left[ \mathop {\mathrm {Re}}\Sigma ^{\prime (2)}_{\phi _1} \right] ^{\text {div}}_{|p^2 = 0}, \end{aligned}$$

10b $$\begin{aligned}&\delta Z_{\mathcal{H}_2}^{(2)} = \delta Z_{\mathcal{H}_2}^{\overline{\mathrm {DR}}(2)} \; = \; - \left[ \mathop {\mathrm {Re}}\Sigma ^{\prime (2)}_{\phi _2} \right] ^{\text {div}}_{|p^2 = 0}, \end{aligned}$$

10c $$\begin{aligned}&\delta \tan \beta ^{(2)}= \delta \tan \beta ^{\overline{\mathrm {DR}}(2)} \; = \; \tfrac{1}{2}\left( \delta Z_{\mathcal{H}_2}^{(2)} - \delta Z_{\mathcal{H}_1}^{(2)} \right) . \end{aligned}$$

The term in Eq. (10c) is in general not the proper expression beyond one-loop order even in the $\overline{\mathrm{{DR}}}$ scheme. For our approximation, however, with only the top Yukawa coupling at the two-loop level, it is the correct $\overline{\mathrm{{DR}}}$ form [70, 71].

The two-loop mass counterterms in the renormalized self-energies (3) are now expressed in terms of the two-loop parameter renormalization constants, determined above, as follows:

11a $$\begin{aligned} \delta m_{\phi _1}^{2(2)}&= \,\delta M_Z^{2(2)}\, \cos ^2 \beta + \delta M_A^{2(2)}\sin ^2 \beta \nonumber \\&\quad - \delta T_1^{(2)}\frac{e}{2 M_Ws_\mathrm {w}} \, \cos \beta (1 + \sin ^2 \beta )\nonumber \\&\quad + \delta T_2^{(2)}\frac{e}{2 M_Ws_\mathrm {w}} \, \cos ^2 \beta \sin \beta \nonumber \\&\quad + 2\, \delta \tan \beta ^{(2)}\, \cos ^2\beta \sin ^2\beta \, (M_A^2 - M_Z^2), \end{aligned}$$

11b $$\begin{aligned} \delta m_{\phi _1\phi _2}^{2(2)}&= - (\delta M_Z^{2(2)}+ \delta M_A^{2(2)}) \sin \beta \cos \beta \nonumber \\&\quad - \delta T_1^{(2)}\frac{e}{2 M_Ws_\mathrm {w}} \, \sin ^3 \beta - \delta T_2^{(2)}\frac{e}{2 M_Ws_\mathrm {w}} \, \cos ^3 \beta \nonumber \\&\quad - \delta \tan \beta ^{(2)}\, \cos \beta \sin \beta \cos 2\beta \, (M_A^2 + M_Z^2), \end{aligned}$$

11c $$\begin{aligned} \delta m_{\phi _2}^{2(2)}&= \delta M_Z^{2(2)}\sin ^2 \beta + \delta M_A^{2(2)}\cos ^2 \beta \nonumber \\&\quad + \delta T_1^{(2)}\frac{e}{2 M_Ws_\mathrm {w}} \, \sin ^2 \beta \cos \beta \nonumber \\&\quad - \delta T_2^{(2)}\frac{e}{2 M_Ws_\mathrm {w}} \, \sin \beta (1 + \cos ^2 \beta )\nonumber \\&\quad - 2\, \delta \tan \beta ^{(2)}\, \cos ^2\beta \sin ^2\beta \, (M_A^2 - M_Z^2). \end{aligned}$$

The *Z*-mass counterterm is again kept for completeness; it does not contribute in the approximation of $\mathcal{O}(\alpha _t\alpha _s)$ considered here.

### Diagram evaluation

Our calculation is performed in the Feynman-diagrammatic (FD) approach. To arrive at expressions for the unrenormalized self-energies and tadpoles at $\mathcal{O}(\alpha _t\alpha _s)$, the evaluation of genuine two-loop diagrams and one-loop graphs with counterterm insertions is required. For the counterterm insertions, described in Sect. 2.4, one-loop diagrams with external top quarks/squarks have to be evaluated as well, as displayed in Fig. 1. The calculation is performed in dimensional reduction [72, 73].

The complete set of contributing Feynman diagrams was generated with the program FeynArts [74–76] (using the model file including counterterms from Ref. [77]), tensor reduction and the evaluation of traces was done with support from the programs FormCalc [78] and TwoCalc [79, 80], yielding algebraic expressions in terms of the scalar one-loop functions $A_0$, $B_0$ [81], the massive vacuum two-loop functions [82], and two-loop integrals which depend on the external momentum. These integrals were evaluated with the program SecDec [64–66], where up to four different masses in 34 different mass configurations needed to be considered, with differences in the kinematic invariants of several orders of magnitude.

### The scalar-top sector of the MSSM

The bilinear part of the top-squark Lagrangian,

12 $$\begin{aligned} \mathcal{L}_{\tilde{t}, \text {mass}}&= - \begin{pmatrix} {{\tilde{t}}_{L}}^{\dagger }, {{\tilde{t}}_{R}}^{\dagger } \end{pmatrix} {\mathbf {M}}_{\tilde{t}}\begin{pmatrix}{\tilde{t}}_{L}\\ {\tilde{t}}_{R} \end{pmatrix} , \end{aligned}$$

contains the stop-mass matrix

13 $$\begin{aligned} {\mathbf {M}}_{\tilde{t}} = \left( \begin{array}{l@{\quad }l} M_{\tilde{t}_L}^2 + m_t^2 + M_Z^2 \cos 2\beta \, (T_t^3 - Q_t s_\mathrm {w}^2) &{} m_tX_t\\ m_tX_t&{} M_{\tilde{t}_R}^2 + m_t^2 + M_Z^2 \cos 2\beta \, Q_t \, s_\mathrm {w}^2 \end{array}\right) , \end{aligned}$$

with

14 $$\begin{aligned} X_t= A_t- \mu \,\cot \beta \end{aligned}$$

where $Q_t$ and $T_t^3$ denote the charge and isospin of the top quark, $A_t$ the trilinear coupling between the Higgs bosons and the scalar tops, and $\mu$ the Higgsino mass parameter. Below we use $M_\mathrm{SUSY}:= M_{\tilde{t}_L}= M_{\tilde{t}_R}$ for our numerical evaluation. The analytical calculation was performed for arbitrary $M_{\tilde{t}_L}$ and $M_{\tilde{t}_R}$, however. ${\mathbf {M}}_{\tilde{t}}$ can be diagonalized with the help of a unitary transformation matrix ${{\mathbf {U}}}_{\tilde{t}}$, parameterized by a mixing angle ${\theta }_{\tilde{t}}$, to provide the eigenvalues $m_{\tilde{t}_1}^2$ and $m_{\tilde{t}_2}^2$ as the squares of the two on-shell top-squark masses.

For the evaluation of the $\mathcal{O}(\alpha _t\alpha _s)$ two-loop contributions to the self-energies and tadpoles of the Higgs sector, renormalization of the top/stop sector at $\mathcal{O}(\alpha _s)$ is required, giving rise to the counterterms for sub-loop renormalization.

We follow the renormalization at the one-loop level given in Refs. [31, 83–85], where details can be found. In particular, in the context of this paper, an OS renormalization is performed for the top-quark mass as well as for the scalar-top masses. This is different from the approach pursued, for example, in Ref. [45], where a $\overline{\mathrm {DR}}$ renormalization was employed, or similarly in the pure $\overline{\mathrm {DR}}$ renormalization presented in Ref. [2]. Using the OS scheme allows us to consistently combine our new correction terms with the hitherto available self-energies included in FeynHiggs.

Besides employing a pure OS renormalization for the top/stop masses in our calculation, we also obtain a result in which the top-quark mass is renormalized $\overline{\mathrm {DR}}$. This new top-quark mass renormalization is included as a new option in the code FeynHiggs. The comparison of the results using the $\overline{\mathrm {DR}}$ and the OS renormalization allows one to estimate (some) missing three-loop corrections in the top/stop sector.

Finally, at $\mathcal{O}(\alpha _t\alpha _s)$, gluinos appear as virtual particles only at the two-loop level (hence, no renormalization for the gluinos is needed). The corresponding soft-breaking gluino mass parameter $M_3$ determines the gluino mass, $m_{\tilde{g}}= M_3$.

### Evaluation and implementation in the program FeynHiggs

The resulting new contributions to the neutral $\mathcal{CP}$-even Higgs-boson self-energies, containing all momentum-dependent and additional constant terms, are assigned to the differences

15 $$\begin{aligned} \Delta \hat{\Sigma }_{ab}(p^2) = \hat{\Sigma }_{ab}^{(2)}(p^2) - \tilde{\Sigma }_{ab}^{(2)}(0), \quad ab = \{HH,hH,hh\}.\nonumber \\ \end{aligned}$$

These are the new terms evaluated in Ref. [1], included in FeynHiggs. Note the tilde (not hat) on $\tilde{\Sigma }^{(2)}(0)$, which signifies that not only the self-energies are evaluated at zero external momentum but also the corresponding counterterms, following Refs. [27–29]. A finite shift $\Delta \hat{\Sigma }(0)$ therefore remains in the limit $p^2\rightarrow 0$ due to $\delta M_A^{2(2)}= \mathop {\mathrm {Re}}\Sigma _{AA}^{(2)}(M_A^2)$ being computed at $p^2=M_A^2$ in $\hat{\Sigma }^{(2)}$, but at $p^2=0$ in $\tilde{\Sigma }^{(2)}$; for details see Eqs. (9) and (11). For the sake of simplicity we will refer to these terms as $\mathcal{O}(p^2 \alpha _t\alpha _s)$ despite the $M_A^2$ dependence.

## Discussion of renormalization schemes

In this section we compare our results for the $\mathcal{O}(p^2 \alpha _t\alpha _s)$ contributions to the MSSM Higgs-boson self-energies, as given in Ref. [1] to the ones presented subsequently in Ref. [2]. We first show analytically the agreement in the Higgs field renormalization in the two calculations and discuss the differences in the $m_t$ renormalizations. We also present some numerical results in both schemes, demonstrating agreement with Ref. [2] once the $\mathcal{O}(\varepsilon )$ terms are dropped from the top-quark mass counterterm.

**Fig. 2:**
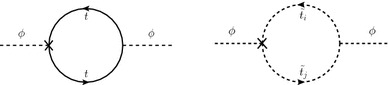
One-loop subrenormalization diagram contributing to $\delta _{\Sigma _{22}}(p^2)$ and $\delta _A(p^2)$, with the counterterm insertion denoted by a *cross*. The *right* diagram only contributes to $\delta _{\Sigma _{22}}(0)$ and $\delta _A(0)$

Using an OS renormalization for the top-quark mass, the counterterm is determined from the components of the $\mathcal{O}(\alpha _s)$ top-quark self-energy (Fig. 1) as follows:

16 $$\begin{aligned} \frac{\delta m_t^\mathrm{OS}}{m_t}&= \frac{1}{2} \mathop {\mathrm {Re}}\left\{ \left[ \Sigma _t^L (m_t^2) + \Sigma _t^R (m_t^2) \right] \right. \nonumber \\&\,\quad \left. + \left[ \Sigma _t^{SL} (m_t^2) + \Sigma _t^{SR} (m_t^2) \right] \right\} , \end{aligned}$$

where the top-quark self-energy is decomposed according to

17 $$\begin{aligned} \Sigma _t (p)&= {p}\!\!\!/\, \omega _{-} \Sigma _t^L (p^2) + {p}\!\!\!/\, \omega _{+} \Sigma _t^R (p^2)\nonumber \\&\quad + m_t\,\omega _{-} \Sigma _t^{SL} (p^2) + m_t\,\omega _{+} \Sigma _t^{SR} (p^2)~. \end{aligned}$$

with the projectors $\omega _{\pm } = \frac{1}{2}(\mathrm{1l}\pm \gamma _5)$.

### Analytical comparison

In the $\mathcal{O}(\alpha _t\alpha _s)$ calculation of the Higgs-boson self-energies the renormalization of the top-quark mass at $\mathcal{O}(\alpha _s)$ is required. The contributing diagrams are shown in the top row of Fig. 1. The top-quark mass counterterm is inserted into the sub-loop renormalization of the two-loop contributions to the Higgs-boson self-energies, where two sample diagrams are shown in Fig. 2. The left diagram contributes to the momentum-dependent two-loop self-energies, while the right one contributes only to the momentum-independent part.

Evaluating the expression in Eq. (16) in $4 - 2\varepsilon$ dimensions yields the OS top-quark mass counterterm at the one-loop level, which can be written as a Laurent expansion in $\varepsilon$,

18 $$\begin{aligned} \delta m_t^\mathrm{OS}= \frac{1}{\varepsilon }\,\delta m_t^\mathrm{div}+ \delta m_t^\mathrm{fin}+ \varepsilon \,\delta m_t^{\varepsilon }+ \cdots ; \end{aligned}$$

higher powers in $\varepsilon$, indicated by the ellipses, do not contribute at the two-loop level for $\varepsilon \rightarrow 0$ after renormalization. Accordingly, the $\overline{\mathrm {DR}}$ top-quark mass counterterm is given by the singular part of Eq. (18),

19 $$\begin{aligned} \delta m_t^{\overline{\mathrm {DR}}}= \frac{1}{\varepsilon }\,\delta m_t^\mathrm{div}. \end{aligned}$$

For further use we define the quantity

20 $$\begin{aligned} \delta m_t^\mathrm{FIN}= \frac{1}{\varepsilon }\,\delta m_t^\mathrm{div}+ \delta m_t^\mathrm{fin}. \end{aligned}$$

At $\mathcal{O}(\alpha _s)$ the OS counterterm is given as

21 $$\begin{aligned} \frac{\delta m_t^\mathrm{OS}}{m_t}&=\frac{\alpha _s}{6 \pi } \, \left\{ - 2\frac{A_0(m_t^2)}{m_t^2}-4\,B_0(m_t^2,0,m_t^2) \right. \nonumber \\&\quad -2 \frac{A_0(m_{\tilde{g}}^2)}{m_t^2} + \frac{A_0(m_{\tilde{t}_1}^2)}{m_t^2} + \frac{A_0(m_{\tilde{t}_2}^2)}{m_t^2} \nonumber \\&\quad + \frac{m_{\tilde{g}}^2 +m_t^2 -m_{\tilde{t}_1}^2 - 4 \,\sin \theta _{\tilde{t}}\cos \theta _{\tilde{t}}\,m_{\tilde{g}}\, m_t}{m_t^2}\nonumber \\&\quad \times \mathop {\mathrm {Re}}\left[ B_0(m_t^2,m_{\tilde{g}}^2,m_{\tilde{t}_1}^2)\right] \nonumber \\&\quad + \frac{m_{\tilde{g}}^2 +m_t^2 -m_{\tilde{t}_2}^2 + 4 \,\sin \theta _{\tilde{t}}\cos \theta _{\tilde{t}}\,m_{\tilde{g}}\, m_t}{m_t^2} \nonumber \\&\left. \quad \times \mathop {\mathrm {Re}}\left[ B_0(m_t^2,m_{\tilde{g}}^2,m_{\tilde{t}_2}^2)\right] \right\} . \end{aligned}$$

The one- and two-point functions $A_0(m^2)$ and $B_0(p^2,m_1^2,m_2^2)$ are expanded in $\varepsilon$ as follows:

22 $$\begin{aligned} A_0(m^2)&= \frac{1}{\varepsilon }\,A_0^{\text {div}}(m^2) + A_0^{\text {fin}}(m^2) + \varepsilon \,A_0^\varepsilon (m^2), \nonumber \\ B_0(p^2,m_1^2,m_2^2)&= \frac{1}{\varepsilon }\,B_0^{\text {div}}(p^2,m_1^2,m_2^2) + B_0^{\text {fin}}(p^2,m_1^2,m_2^2) \nonumber \\&\quad + \varepsilon \,B_0^\varepsilon (p^2,m_1^2,m_2^2)~. \end{aligned}$$

Consequently, the term at $\mathcal{O}(\varepsilon )$, $\delta m_t^{\varepsilon }/m_t$, is given by Eq. (21), but taking only into account the pieces $\propto A_0^\varepsilon ,\, B_0^{\varepsilon }$. The special cases of $A_0^\varepsilon (m^2)$ and $B_0^\varepsilon (m^2,0,m^2)$ are given by

23 $$\begin{aligned}&A_0^\varepsilon (m^2) = m^2\left\{ 1-\log (m^2/\mu ^2)+\frac{1}{2}\log ^2(m^2/\mu ^2) + \frac{\pi ^2}{12}\right\} ,\nonumber \\&B_0^\varepsilon (m^2,0,m^2) = 4 -2\,\log (m^2/\mu ^2)+\frac{1}{2}\log ^2(m^2/\mu ^2) + \frac{\pi ^2}{12}, \end{aligned}$$

where the factor $4\pi \mathrm {e}^{-\gamma _E}$ is absorbed into the renormalization scale. The expression for $B_0^{\varepsilon }$ depending on three mass scales can be found e.g. in Ref. [86].

In our calculation in Ref. [1] we include terms up to $\mathcal{O}(\varepsilon )$, originating from the top-quark self-energy, in the top-mass counterterm,^3^ i.e.

24 $$\begin{aligned} \delta m_t^{{[1]}} = \delta m_t^\mathrm{OS}. \end{aligned}$$

The derivation in Ref. [2] proceeds differently. The renormalized Higgs-boson self-energies are first calculated in a pure $\overline{\mathrm {DR}}$ scheme. This concerns the top mass, the scalar-top masses, the Higgs field renormalization, and $\tan \beta$. In this way it is ensured that in particular the Higgs fields are renormalized using $\overline{\mathrm {DR}}$, $\delta Z_{\mathcal{H}_i} = \delta Z_{\mathcal{H}_i}^{\overline{\mathrm {DR}}}$, where this quantity contains the contribution from the one- and two-loop level. Using this pure $\overline{\mathrm {DR}}$ scheme a finite result is obtained in which all poles in $1/\varepsilon$ and $1/\varepsilon ^2$ cancel, such that the limit $\varepsilon \rightarrow 0$ can be taken. Subsequently, the $\overline{\mathrm {DR}}$ top-quark mass counterterm, $\delta m_t^{\overline{\mathrm {DR}}}$, is replaced by an on-shell counterterm, and the top-quark mass definition is changed accordingly. The same procedure is applied for the scalar-top masses. Since these finite expressions for the renormalized Higgs-boson self-energies do not contain any term of $\mathcal{O}(1/\varepsilon )$, the $\delta m_t^{\varepsilon }$ part of the OS top-quark mass counterterm does not contribute, i.e.

25 $$\begin{aligned} \delta m_t^{{[2]}} = \delta m_t^\mathrm{FIN}. \end{aligned}$$

The numerical results for the renormalized Higgs-boson self-energies obtained this way differ significantly from the ones obtained in Ref. [1], as pointed out in Ref. [2].

In the following we discuss the different Higgs-boson field renormalizations, where we use the notation of $\delta Z_{\mathcal{H}_2}^{\delta m_t^{\text {X}}}$ for the field renormalization derived using $\delta m_t^{\text {X}}$, with $\text {X} = \overline{\mathrm {DR}}$, FIN, $\mathrm {OS}$. The field renormalization can be decomposed into one-loop, two-loop, ...parts as

26 $$\begin{aligned} \delta Z_{\mathcal{H}_2}^{\delta m_t^{\text {X}}} = \delta Z_{\mathcal{H}_2}^{\delta m_t^{\text {X}} (1)} + \delta Z_{\mathcal{H}_2}^{\delta m_t^{\text {X}} (2)} + \cdots \end{aligned}$$

In Ref. [2] it was claimed that using an OS top-quark mass renormalization from the start results in a non-$\overline{\mathrm {DR}}$ renormalization of $\delta Z_{\mathcal{H}_2}$. While it is correct that an OS value for $m_t$ yields different results in the one- and two-loop part,

27 $$\begin{aligned} \delta Z_{\mathcal{H}_2}^{\delta m_t^\mathrm{OS}(1)} \ne \delta Z_{\mathcal{H}_2}^{\delta m_t^{\overline{\mathrm {DR}}}(1)}, \quad \delta Z_{\mathcal{H}_2}^{\delta m_t^\mathrm{OS}(2)} \ne \delta Z_{\mathcal{H}_2}^{\delta m_t^{\overline{\mathrm {DR}}}(2)}, \end{aligned}$$

the sum of the one- and two-loop parts are identical, independently of the choice of the top-quark mass renormalization (see e.g. Eqs. (3.60)–(3.62) in Ref. [90]),

28 $$\begin{aligned} \left( \delta Z_{\mathcal{H}_2}^{{[1]}} = \right) \; \delta Z_{\mathcal{H}_2}^{\delta m_t^\mathrm{OS}}\Big |_{\text {div}}&= \delta Z_{\mathcal{H}_2}^{\delta m_t^\mathrm{FIN}} = \delta Z_{\mathcal{H}_2}^{\delta m_t^{\overline{\mathrm {DR}}}} \;\left( = \delta Z_{\mathcal{H}_2}^{{[2]}} \right) , \end{aligned}$$

provided that also in $\delta Z_{\mathcal{H}_2}^{\delta m_t^\mathrm{OS}}$ all finite pieces are dropped, as done in Ref. [1]. Differences between $\delta Z_{\mathcal{H}_2}^{{[1]}}$ and $\delta Z_{\mathcal{H}_2}^{{[2]}}$ arise only at the three-loop level. Consequently, the claim in Ref. [2] that using $\delta m_t^\mathrm{OS}$ leads to an inconsistency in the Higgs field renormalization in Ref. [1] is not correct. The field renormalizations thus cannot be responsible for the observed differences between Refs. [1, 2].

More explicitly, the difference between the two calculations results from non-vanishing $\delta m_t^{\varepsilon }$ terms in the renormalized Higgs-boson self-energies. Those terms naturally appear when performing a full expansion in the dimensional regulator $\varepsilon$. The latter corresponds to choosing $\delta m_t^\mathrm{OS}$ (as done in Ref. [1]) instead of $\delta m_t^\mathrm{FIN}$ (as done in Ref. [2]).

In order to isolate the contributions coming from $$\mathcal{O}(\varepsilon )$$ terms $$\times $$$$1/\varepsilon $$ poles we define the following quantities, where superscripts $$\mathrm {OS}$$, $$\mathrm {FIN}$$ refer to the respective use of $$\delta m_t^\mathrm{OS}$$, $$\delta m_t^\mathrm{FIN}$$:

29a $$\begin{aligned}&\delta T_i^{(2)\mathrm {OS}} = \delta T_i^{(2)\,\mathrm {FIN}} + \delta _{T_i}~, \end{aligned}$$

29b $$\begin{aligned}&\Sigma _{\phi _{ij}}^{(2)\mathrm {OS}}(p^2) = \Sigma _{\phi _{ij}}^{(2)\,\mathrm {FIN}}(p^2) + \delta _{\Sigma _{ij}}(p^2)~, \end{aligned}$$

29c $$\begin{aligned}&\Sigma _{AA}^{(2)\mathrm {OS}}(p^2) = \Sigma _{AA} ^{(2)\,\mathrm {FIN}} (p^2) + \delta _A(p^2)~, \end{aligned}$$

where the last equation yields a shift for the *A*-boson mass counterterm in Eq. (7),

30 $$\begin{aligned} \delta M_A^{2(2)\mathrm {OS}} = \delta M_A^{2(2)\,\mathrm {FIN}}+\delta _A(M_A^2). \end{aligned}$$

**Fig. 3:**
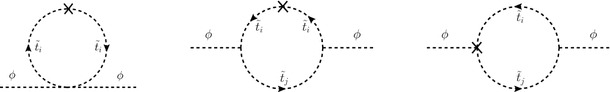
One-loop subrenormalization diagrams containing top-squark loops with counterterm insertions

The $\delta$-terms are defined as the *finite* contributions stemming from $\delta m_t^{\varepsilon }$-dependent parts in the counterterms (see the left diagram in Fig. 2 for an example). The $\overline{\mathrm {DR}}$-renormalized quantities do not contain a finite $\delta m_t^{\varepsilon }$-dependent part by definition. Furthermore, since $\phi _1$ has no coupling to the top quark, there are no terms proportional to $\delta m_t^{\varepsilon }$ in $\Sigma _{\phi _1}^{(2)}$, $\Sigma _{\phi _1\phi _2}^{(2)}$, and $\delta T_1^{(2)}$, and it is sufficient to consider $\delta _{\Sigma _{22}}$, $\delta _{A}$, and $\delta _{T_2}$ only. While $\delta _{T_2}$ is $p^2$-independent, we find

31 $$\begin{aligned} \delta _{\Sigma _{22}}(p^2)&= \frac{3 \alpha _t}{2 \pi } \,p^2 \frac{\delta m_t^{\varepsilon }}{m_t} +\delta _{\Sigma _{22}}(0), \end{aligned}$$

32 $$\begin{aligned} \delta _{A}(p^2)&= \frac{3 \alpha _t}{2 \pi } \,p^2 \cos ^2 \beta \, \frac{\delta m_t^{\varepsilon }}{m_t} +\delta _{A}(0). \end{aligned}$$

Using Eqs. (3), (11) we find that the following relations hold for the renormalized Higgs-boson self-energies:

33 $$\begin{aligned}&- \sin ^2 \beta \,\delta _A(0) - \frac{e}{2 M_Ws_\mathrm {w}} \, \cos ^2 \beta \sin \beta \, \delta _{T_2} = 0 \quad (\text {for }\hat{\Sigma }_{\phi _1}^{(2)}), \nonumber \\&\sin \beta \cos \beta \, \delta _A(0) + \frac{e}{2 M_Ws_\mathrm {w}} \, \cos ^3 \beta \, \delta _{T_2} = 0 \quad (\text {for }\hat{\Sigma }_{\phi _1\phi _2}^{(2)}), \nonumber \\&\delta _{\Sigma _{22}}(0) - \cos ^2 \beta \,\delta _A(0) + \frac{e}{2 M_Ws_\mathrm {w}} \, \sin \beta (1 + \cos ^2 \beta )\, \delta _{T_2} \nonumber \\&\quad =\; 0 \quad (\text {for }\hat{\Sigma }_{\phi _2}^{(2)}). \end{aligned}$$

This is in agreement with the observation that in the renormalized Higgs-boson self-energies at zero external momentum at $\mathcal{O}(\alpha _t\alpha _s)$, the terms containing $\delta m_t^{\varepsilon }$ drop out in the final (finite) result. Such a cancellation is to be expected as the same combination of one-loop self-energies that potentially contributes to this finite contribution also appears in the $\mathcal{O}(1/\varepsilon )$ term, where they must cancel. This argument in principle still holds when the momentum-dependent $\mathcal{O}(\alpha _t\alpha _s)$ corrections are calculated and *all* counterterms are evaluated with a full expansion in $\varepsilon$. Since the counterterm $\delta _A$ is evaluated at $p^2=M_A^2$, and the Higgs-boson fields are renormalized in the $\overline{\mathrm {DR}}$ scheme, however, one finds, using Eqs. (3) and (11) for the three renormalized Higgs-boson self-energies,

34 $$\begin{aligned}&- \sin ^2 \beta \, \left( \delta _A(M_A^2) - \delta _A(0) \right) \nonumber \\&\quad = \frac{3 \alpha _t}{2 \pi } \left( - \cos ^2\beta \,\sin ^2\beta \,M_A^2 \right) \frac{\delta m_t^{\varepsilon }}{m_t}\quad \left( \text{ for } \hat{\Sigma }_{\phi _1}^{(2)}\right) , \nonumber \\&\sin \beta \cos \beta \, \left( \delta _A(M_A^2) - \delta _A(0) \right) \nonumber \\&\quad = \frac{3 \alpha _t}{2 \pi } \left( + \cos ^3\beta \,\sin \beta \,M_A^2 \right) \frac{\delta m_t^{\varepsilon }}{m_t}\quad \left( \text{ for } \hat{\Sigma }_{\phi _1\phi _2}^{(2)}\right) , \nonumber \\&\left( \delta _{\Sigma _{22}}(p^2) - \delta _{\Sigma _{22}}(0) \right) - \cos ^2 \beta \, \left( \delta _A(M_A^2) - \delta _A(0) \right) \nonumber \\&\quad = \frac{3 \alpha _t}{2 \pi } \left( p^2 - \cos ^4\beta \,M_A^2 \right) \frac{\delta m_t^{\varepsilon }}{m_t} \quad \left( \text{ for } \hat{\Sigma }_{\phi _2}^{(2)}\right) , \end{aligned}$$

i.e. the $\delta m_t^{\varepsilon }$ terms contribute in the newly evaluated $\mathcal{O}(p^2 \alpha _t\alpha _s)$ corrections. They are $p^2$-independent in $\hat{\Sigma }_{\phi _1}^{(2)}$ and $\hat{\Sigma }_{\phi _1\phi _2}^{(2)}$, while they do depend on $p^2$ in $\hat{\Sigma }_{\phi _2}^{(2)}$.

The $p^2$-dependent terms coming from the expansion of terms like $(-p^2)^{-\varepsilon }$ multiplying a $1/\varepsilon ^2$ divergence must certainly cancel after inclusion of the counterterms, because non-local terms cannot appear in a renormalizable theory. However, the cancellation of the $\varepsilon$-dependent terms stemming from the mass renormalization is not necessarily fulfilled once the two-loop amplitude carries full momentum dependence. Similarly, the truncation of the field renormalization to the divergent part cuts away terms involving $\delta m_t^{\varepsilon }$, leading to further non-cancellations. The explicit $\overline{\mathrm {DR}}$ renormalization of the Higgs-boson fields drops the corresponding finite contributions, such that no $\delta m_t^\mathrm{fin}$, $\delta m_t^{\varepsilon }$ terms are taken into account. The different dependence on the external momentum and the $\overline{\mathrm {DR}}$ prescription for the Higgs field renormalization leads to Eqs. (34).

Equivalent momentum-dependent terms of $\mathcal{O}(\varepsilon )$ of the scalar-top mass counterterms, evaluated from the diagrams in the lower row of Fig. 1, do not contribute. The diagrams with top-squark counterterm insertions are depicted in Fig. 3. The first diagram is momentum independent. In the second diagram, the corresponding loop integral is a massive scalar three-point function ($C_0$) with only scalar particles running in the loop, and thus is UV finite. Consequently, the top-squark mass counterterm insertions of $\mathcal{O}(\varepsilon )$ do not contribute. In the third diagram the stop mass counterterm can enter via the (dependent) counterterm for $A_t$ [29, 84]. This diagram does not possess a momentum-dependent divergence, however, and thus the $\mathcal{O}(\varepsilon )$ term of the scalar-top mass counterterm again does not contribute.

### Physics content and interpretation

In the following we give another view on the finite $\delta m_t^{\varepsilon }$ term from the top mass renormalization and on the interpretation of the different results for the Higgs-boson masses with and without this term.

In the approximation with $p^2=0$ for the two-loop self-energies, the results are the same for either dropping or including the $\delta m_t^{\varepsilon }$ term, provided that this is done everywhere in the contributions from the top–stop sector in the renormalized two-loop self-energies.

As explained above, abandoning the $p^2=0$ approximation yields an additional $\delta m_t^{\varepsilon }$ in the $p^2$-coefficient of the self-energy $\Sigma _{\phi _2}^{(2)} (p^2)$ when the on-shell top-quark mass counterterm, see Eq. (18), is used, as well as in the *A*-boson self-energy $\Sigma _{AA}(p^2)$ from which it induces an additive term ${\sim }M_A^2\, \delta m_t^{\varepsilon }/m_t$ to the mass counterterm $\delta M_A^2$.

In the renormalized self-energy $\hat{\Sigma }_{\phi _2}^{(2)} (p^2)$, Eq. (3c), this extra $p^2$-dependent term survives when $\delta Z_{\mathcal{H}_2}^{(2)}$ is defined in the minimal way containing only the $1/\varepsilon$ and $1/\varepsilon ^2$ singular parts; however, it disappears in $\hat{\Sigma }_{\phi _2}^{(2)} (p^2)$ when the minimal $\delta Z_{\mathcal{H}_2}^{(2)}=\delta Z_{\mathcal{H}_2}^{\delta m_t^\mathrm{OS}\,(2)}\Big |_\mathrm{div}$ is replaced by

35 $$\begin{aligned} \delta Z_{\mathcal{H}_2}^{(2)} \rightarrow \delta Z_{\mathcal{H}_2}^{(2)} - \frac{3\alpha _t}{2\pi } \frac{\delta m_t^{\varepsilon }}{m_t} , \end{aligned}$$

which now accommodates also a finite part of two-loop order.

This shift in $\delta Z_{\mathcal{H}_2}^{(2)}$ by a finite term has also an impact on the counterterm for $\tan \beta$ via $\delta \tan \beta = \frac{1}{2} \delta Z_{\mathcal{H}_2}^{(2)}$. This has the consequence that the extra $\delta m_t^{\varepsilon }$ term in $\delta M_A^2$ drops out in the constant counterterms for the renormalized self-energies $\hat{\Sigma }_{\phi _{ij}}^{(2)} (p^2)$ in Eq. (3) because of cancellations with the $\delta m_t^{\varepsilon }$ term in $\delta \tan \beta$ and $\delta Z_{\mathcal{H}_2}^{(2)}$ [this can be seen from the explicit expressions given in Eqs. (6) and (11)].

Accordingly, keeping or dropping the finite $\delta m_t^{\varepsilon }$ part is thus equivalent to a finite shift in the field-renormalization constant $\delta Z_{\mathcal{H}_2}$ at the two-loop level, which corresponds to a finite shift in $\tan \beta$ as input quantity. Numerically, the shift in $\tan \beta$ is small, and cannot explain the differences in the $M_h$ predictions from the two schemes. Hence, these differences originate from the different $p^2$ coefficients in $\hat{\Sigma }_{\phi _2}^{(2)} (p^2)$.

The impact of a modification of the two-loop field-renormalization constant on the mass $M_h$ can best be studied in terms of the self-energy $\Sigma _{hh}$ in the *h*, *H* basis, which is composed of the $\Sigma _{\phi _{ij}}$ in the following way:

36 $$\begin{aligned} \Sigma _{hh} = \, \cos ^2\alpha \, \Sigma _{\phi _2} + \sin ^2\alpha \, \Sigma _{\phi _1} -2\sin \alpha \cos \alpha \, \Sigma _{\phi _1\phi _2} , \end{aligned}$$

where only $\Sigma _{\phi _2}$ contains the $p^2$-dependent $\delta m_t^{\varepsilon }$ contribution. In order to simplify the discussion and to point to the main features, we assume sufficiently large values of $\tan \beta$, such that we can write $\hat{\Sigma }_{hh} \simeq \,\hat{\Sigma }_{\phi _2}$, and *h*, *H* mixing effects play only a marginal role (both simplifications apply to the numerical discussions in the subsequent section). Moreover, to simplify the notation, we drop the indices and define

37 $$\begin{aligned} \Sigma _{hh} \equiv \Sigma _{} , \quad \hat{\Sigma }_{hh} \equiv \hat{\Sigma }_{} , \quad \delta Z_{hh} \equiv \delta Z , \end{aligned}$$

where $\delta Z_{hh} = \cos ^2\alpha \, \delta Z_{\mathcal{H}_2} + \sin ^2 \alpha \, \delta Z_{\mathcal{H}_1} \simeq \delta Z_{\mathcal{H}_2}$. Starting from the tree-level mass $m_h$ and the renormalized *h* self-energy up to the two-loop level,

38 $$\begin{aligned} \hat{\Sigma }_{} (p^2) = \, \Sigma _{} (p^2) -\delta m_h^2 +\delta Z (p^2 - m_h^2), \end{aligned}$$

we obtain the higher-order corrected mass $M_h$ from the pole of the propagator, i.e.

39 $$\begin{aligned} M_h^2 - m_h^2 + \hat{\Sigma }_{} (M_h^2) = 0 . \end{aligned}$$

The Taylor-expansion of the unrenormalized self-energy around $p^2=0$,

40 $$\begin{aligned} \Sigma _{} (p^2) = \, \Sigma _{} (0) + p^2 \, \Sigma _{}' (0) + \tilde{\Sigma _{}} (p^2), \end{aligned}$$

yields the first two terms containing the singularities in $1/\varepsilon$ and $1/\varepsilon ^2$, and the residual fully finite and scheme-independent part denoted by $\tilde{\Sigma _{}} (p^2)$. With this expansion inserted into Eq. (38) one obtains from the pole condition Eq. (39) the relation

41 $$\begin{aligned}&(M_h^2 - m_h^2) \left[ 1 + \delta Z + \Sigma _{}'(0) \right] + \left[ \Sigma _{}(0) - \delta m_h^2 + m_h^2 \, \Sigma _{}'(0) \right] \nonumber \\&\quad + \, \tilde{\Sigma _{}} (M_h^2) = \, 0 , \end{aligned}$$

where the expressions in the square brackets are each finite, irrespective of a possible finite term in the definition of $\delta Z$.

Taking into account that $M_h^2$ differs from $m_h^2$ by a higher-order shift, we can replace

42 $$\begin{aligned} \tilde{\Sigma _{}} (M_h^2) = \, \tilde{\Sigma _{}} (m_h^2) + (M_h^2 -m_h^2)\, \tilde{\Sigma _{}}' (m_h^2) + \cdots \end{aligned}$$

and obtain

43 $$\begin{aligned} M_h^2 - m_h^2&= \, - \frac{\Sigma _{}(0) -\delta m_h^2 +m_h^2\, \Sigma _{}'(0) + \tilde{\Sigma _{}}(m_h^2) }{1+\delta Z + \Sigma _{}'(0) + \tilde{\Sigma _{}}'(m_h^2) } \nonumber \\ \nonumber&= \, - \big [ \Sigma _{}(0) -\delta m_h^2 +m_h^2\, \Sigma _{}'(0) +\tilde{\Sigma _{}}(m_h^2) \big ]_\mathrm{1loop\,+\,2loop} \\ \nonumber&\quad + \big [ \Sigma _{}(0) -\delta m_h^2 +m_h^2\, \Sigma _{}'(0) + \tilde{\Sigma _{}}(m_h^2)\big ]_\mathrm{1loop} \\&\quad \cdot \big [\delta Z + \Sigma _{}'(0) + \tilde{\Sigma _{}}'(m_h^2)\big ]_\mathrm{1loop} + \, \cdots \end{aligned}$$

showing explicitly all terms up to two-loop order. It does not contain the two-loop part of the field-renormalization constant, which indeed would show up at the three-loop level. Hence, effects resulting from different conventions for $\delta Z^{(\mathrm{2loop})}$ in the finite part have to be considered in the current situation as part of the theoretical uncertainty.

### Numerical comparison

In this section the renormalized momentum-dependent $\mathcal{O}(p^2 \alpha _t\alpha _s)$ self-energy contributions $\Delta \hat{\Sigma }_{hh}$, $\Delta \hat{\Sigma }_{hH}$, $\Delta \hat{\Sigma }_{HH}$ of Eq. (15) and the mass shifts

44 $$\begin{aligned} \Delta M_h= M_h- M_{h,0}, \quad \Delta M_H= M_H- M_{H,0} \end{aligned}$$

are compared using either $\delta m_t^\mathrm{OS}$ or $\delta m_t^\mathrm{FIN}$, as discussed above. $M_{h,0}$ and $M_{H,0}$ denote the Higgs-boson mass predictions *without* the newly obtained $\mathcal{O}(p^2 \alpha _t\alpha _s)$ corrections.

The results are obtained for two different scenarios. Scenario 1 is adopted from the $m_h^\mathrm{max}$ scenario described in Ref. [91]. We use the following parameters:

45 $$\begin{aligned} m_t&= 173.2\,\, \mathrm {GeV},\quad M_\mathrm{SUSY}=1\,\, \mathrm {TeV},\quad X_t=2\,M_\mathrm{SUSY}, \nonumber \\ m_{\tilde{g}}&= 1500\,\, \mathrm {GeV},\quad \mu = M_2 = 200\,\, \mathrm {GeV}. \end{aligned}$$

Here $M_2$ denotes the *SU*(2) soft SUSY-breaking parameter, where the *U*(1) parameter is derived via the GUT relation $M_1 = (5/3)\, (s_\mathrm {w}^2/c_\mathrm {w}^2)\, M_2$. Scenario 2 is an updated version of the “light-stop scenario” of Refs. [91, 92]

46 $$\begin{aligned} m_t&= 173.2\,\, \mathrm {GeV},\quad M_\mathrm{SUSY}= 0.5 \,\, \mathrm {TeV},\quad X_t=2\,M_\mathrm{SUSY}, \nonumber \\ m_{\tilde{g}}&= 1500\,\, \mathrm {GeV},\quad \mu = M_2 = 400\,\, \mathrm {GeV}\; M_1 = 340 \,\, \mathrm {GeV}, \end{aligned}$$

leading to stop mass values of

47 $$\begin{aligned} m_{\tilde{t}_1}= 326.8 \,\, \mathrm {GeV}, \quad m_{\tilde{t}_2}= 673.2 \,\, \mathrm {GeV}. \end{aligned}$$

A renormalization scale of $\mu = m_t$ is set in all numerical evaluations.

### Self-energies

**Fig. 4:**
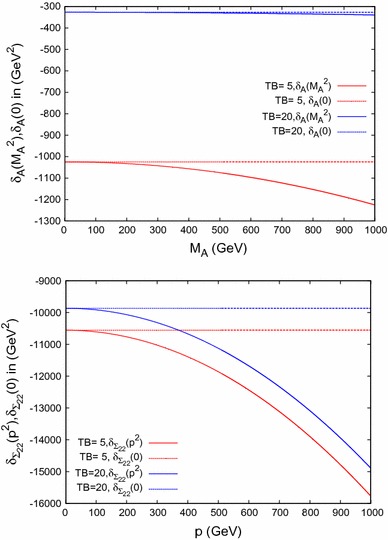
$\delta _{A}(M_A^2)$ and $\delta _{A}(0)$ varying $M_A$ shown in the *upper plot*, $\delta _{\Sigma _{22}}(p^2)$ and $\delta _{\Sigma _{22}}(0)$ in the *lower plot*, both within Scenario 1

In Fig. 4 we present the results for the $\delta _A$ (upper plot) and $\delta _{\Sigma _{22}}$ (lower plot) contributions for $\tan \beta = 5 (20)$ in red (blue) in Scenario 1, where $\delta _A,\delta _{\Sigma _{22}}$ are defined in Eqs. (31) and (32). In the upper plot $\delta _A(M_A^2)$ ($\delta _A(0)$) is shown as solid (dashed) line; correspondingly, in the lower plot $\delta _{\Sigma _{22}}(p^2)$ ($\delta _{\Sigma _{22}}(0)$) is depicted as solid (dashed) line. The contribution is seen to decrease quadratically with $M_A$ or $p\ (:= {\sqrt{p^2}})$ when including the momentum-dependent terms; see Eq. (34). For $\delta _A$ it is suppressed with $\tan ^2 \beta$. For high values of $M_A$ and low $\tan \beta$, the $\delta _A$ contribution becomes sizable. Similarly, for large *p* the $\delta _{\Sigma _{22}}$ term becomes sizable, showing the relevance of the $\delta m_t^{\varepsilon }$ contribution.

**Fig. 5:**
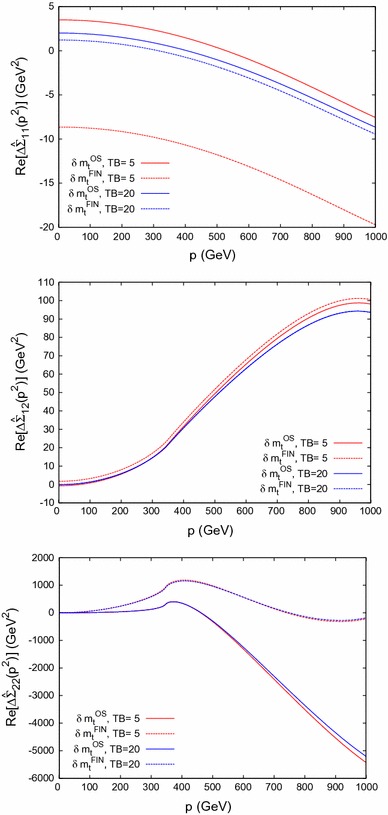
$\Delta \hat{\Sigma }_{\phi _{ij}}$ in Scenario 1 (with $M_A=250 \,\, \mathrm {GeV}$) for $ij = 11, 12, 22$ in the *upper*, the *middle* and the *lower plot*, respectively. The *solid* (*dashed*) *lines* show the result obtained with $\delta m_t^\mathrm{OS}$ ($\delta m_t^\mathrm{FIN}$); the *red* (*blue*) *lines* correspond to $\tan \beta = 5 (20)$

The behavior of the real parts of the two-loop contributions to the self-energies $\Delta \hat{\Sigma }_{ab}$ is analyzed in Fig. 5. Solid lines show the result evaluated with $\delta m_t^\mathrm{OS}$, as obtained in Ref. [1] [i.e. the new contribution added to the previous FeynHiggs result in Ref. [1]; see Eq. (15)]. Dashed lines show the result evaluated with $\delta m_t^\mathrm{FIN}$, as obtained in Ref. [2]. We show $M_A= 250 \,\, \mathrm {GeV}$ and $\tan \beta = 5 (20)$ as red (blue) lines. The difference between the $\delta m_t^\mathrm{FIN}$ and $\delta m_t^\mathrm{OS}$ calculations for $\Delta \hat{\Sigma }_{\phi _1}$ and $\Delta \hat{\Sigma }_{\phi _1\phi _2}$ is *p*-independent, as discussed below Eq. (34), and the difference between the two schemes is numerically small. For $\Delta \hat{\Sigma }_{\phi _2}$, on the other hand, the difference becomes large for large values of *p*. This self-energy contribution is mostly relevant for the light $\mathcal{CP}$-even Higgs boson, however, i.e. for $p \sim M_h$, and thus the *relevant* numerical difference remains relatively small (but non-zero) compared to the larger differences at large *p*.

For completeness it should be mentioned that the imaginary part is not affected by the variation of the top-quark renormalization, as only the real parts of the counterterm insertions enter the calculation.

Scenario 2 was omitted as the relevant aspects for the analysis of the self-energies using $\delta m_t^\mathrm{OS}$ vs. $\delta m_t^\mathrm{FIN}$ have become sufficiently apparent within Scenario 1.

### Mass shifts

**Fig. 6:**
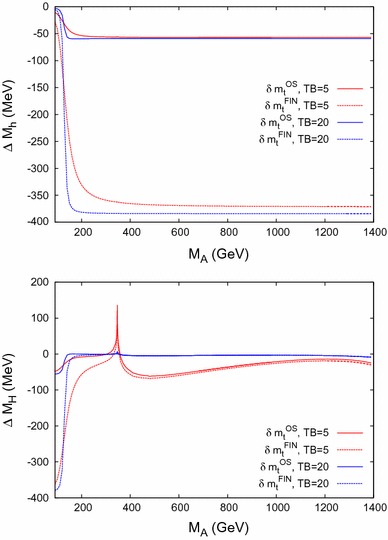
Variation of the mass shifts $\Delta M_h,\Delta M_H$ with the *A*-boson mass $M_A$ within Scenario 1, for $\tan \beta =5$ (*red*) and $\tan \beta = 20$ (*blue*) including or excluding some $\delta$ terms. The peak in $\Delta M_H$ originates from a threshold at $2\,m_t$

We now turn to the effects on the neutral $\mathcal{CP}$-even Higgs-boson masses themselves. The numerical effects on the two-loop corrections to the Higgs-boson masses $M_{h,H}$ are investigated by analyzing the mass shifts $\Delta M_h$ and $\Delta M_H$ of Eq. (44). The results are shown for the two renormalization schemes for the top-quark mass, i.e. using $\delta m_t^\mathrm{OS}$ or $\delta m_t^\mathrm{FIN}$. The color coding is as in Fig. 5. The results for Scenario 1 are shown in Fig. 6 and are in agreement with Figs. 2 and 3 (left) in Ref. [2], i.e. we reproduce the results of Ref. [2] using $\delta m_t^\mathrm{FIN}$.

**Fig. 7:**
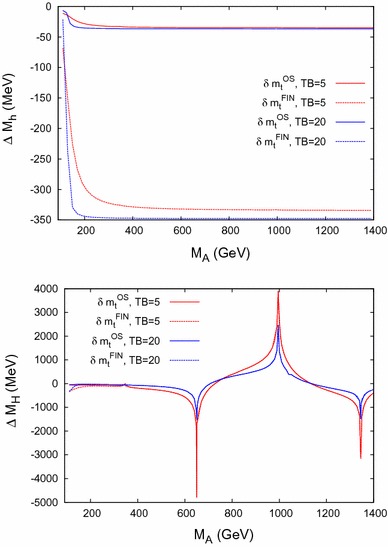
Variation of the mass shifts $\Delta M_h,\Delta M_H$ with the *A*-boson mass $M_A$ within Scenario 2, for $\tan \beta =5$ (*red*) and $\tan \beta = 20$ (*blue*) including or excluding some $\delta$ terms. The peaks in $\Delta M_H$ originate from thresholds at $2\,m_t$, $2\,m_{\tilde{t}_1}$, $m_{\tilde{t}_1}+ m_{\tilde{t}_2}$, and $2\,m_{\tilde{t}_2}$, where the threshold at $2\,m_t$ is suppressed by $1/\tan ^2\beta$

The results for Scenario 2 are shown in Fig. 7. The results are again in agreement with Figs. 2 and 3 (right) in Ref. [2]. This agreement confirms the use of $\delta m_t^\mathrm{FIN}$ in Ref. [2], in comparison with $\delta m_t^\mathrm{OS}$ used in the evaluation of our results.

For the contribution to $M_H$, peaks can be observed at $M_A= 2 m_{\tilde{t}_1}$, $m_{\tilde{t}_1}+ m_{\tilde{t}_2}$, $2 m_{\tilde{t}_2}$; see also Ref. [1] and the discussion of Fig. 9 below.

Since the results using $\delta m_t^\mathrm{OS}$ and $\delta m_t^\mathrm{FIN}$ correspond to two different renormalization schemes, their difference should be regarded as an indication of missing higher-order momentum-dependent corrections.

## Comparison with the $$\varvec{m_t}$$$$\overline{\mathrm {DR}}$$ renormalization

Having examined the renormalization of the top-quark mass, we will now analyze the numerical differences between an $m_t^{\overline{\mathrm {DR}}}$ and an $m_t^{\mathrm {OS}}$ calculation. This has been realized by employing a $\overline{\mathrm {DR}}$ renormalization of the top-quark mass in all steps of the calculation. The top-squark masses are kept renormalized on-shell. This can be seen as an intermediate step toward a full $\overline{\mathrm {DR}}$ analysis.

### Implementation in the program FeynHiggs

In the $\overline{\mathrm {DR}}$ scheme the top-quark mass parameter entering the calculation is the MSSM $\overline{\mathrm {DR}}$ top-quark mass, which at one-loop order is related to the pole mass $m_t$ (given in the user input) in the following way:

48 $$\begin{aligned} m_t^{\overline{\mathrm {DR}}}(\mu ) = m_t\cdot \left[ 1 + \frac{\delta m_t^\mathrm{fin}}{m_t} + \mathcal {O}\left( \bigl (\alpha _s^{\overline{\mathrm {DR}}}\bigr )^2\right) \right] . \end{aligned}$$

The term $\delta m_t^\mathrm{fin}$ can be obtained from Eq. (18), with the formal replacement $\alpha _s \rightarrow \alpha _s^{\overline{\mathrm {DR}}}(\mu )$, yielding

49 $$\begin{aligned} \nonumber \frac{\delta m_t^\mathrm{fin}}{m_t}&= \alpha _s^{\overline{\mathrm {DR}}}(\mu ) \left( -\frac{5}{3\pi } + \frac{1}{\pi }\log (m_t^2/\mu ^2)\right. \\&\quad + \frac{m_{\tilde{g}}^2}{3 m_t^2 \pi } \left( -1 + \log (m_{\tilde{g}}^2/\mu ^2)\right) \nonumber \\ \nonumber&\quad + \frac{1}{6 m_t^2 \pi } \left( m_{\tilde{t}_1}^2 (1 - \log (m_{\tilde{t}_1}^2/\mu ^2))\right. \\&\quad + m_{\tilde{t}_2}^2 (1 - \log (m_{\tilde{t}_2}^2/\mu ^2)) \nonumber \\ \nonumber&\quad + (m_{\tilde{g}}^2 + m_t^2 - m_{\tilde{t}_1}^2 - 2 m_{\tilde{g}}m_t\sin (2 \theta _t))\\&\quad \times \mathop {\mathrm {Re}}[B_0^\mathrm{fin}(m_t^2, m_{\tilde{g}}^2, m_{\tilde{t}_1}^2)]\nonumber \\&\quad + (m_{\tilde{g}}^2 + m_t^2 - m_{\tilde{t}_2}^2 + 2 m_{\tilde{g}}m_t\sin (2 \theta _t))\nonumber \\&\quad \left. \left. \times \mathop {\mathrm {Re}}[B_0^\mathrm {fin}(m_t^2, m_{\tilde{g}}^2, m_{\tilde{t}_2}^2)] \right) \right) . \end{aligned}$$

At zeroth order, $\alpha _s^{\overline{\mathrm {DR}}}(\mu )=\alpha _s^{\overline{\mathrm {MS}}}(\mu )$.

As on-shell renormalized quantities the stop masses $m_{\tilde{t}_1}$ and $m_{\tilde{t}_2}$ should have fixed values, independently of the renormalization chosen for the top-quark mass. We compensate for the changes induced by $\delta m_t^\mathrm{fin}$ in the stop mass matrix, Eq. (13), by shifting the SUSY-breaking parameters as follows:

50a $$\begin{aligned}&M_{\tilde{t}_L}^2 \rightarrow M_{\tilde{t}_L}'^2 = M_{\tilde{t}_L}^2 + (m_t^{\mathrm {OS}})^2 - (m_t^{\overline{\mathrm {DR}}})^2 ,\end{aligned}$$

50b $$\begin{aligned}&7M_{\tilde{t}_R}^2 \rightarrow M_{\tilde{t}_R}'^2 = M_{\tilde{t}_R}^2 + (m_t^{\mathrm {OS}})^2 - (m_t^{\overline{\mathrm {DR}}})^2 ,\end{aligned}$$

50c $$\begin{aligned}&A_t\rightarrow A_t' = \frac{m_t^{\mathrm {OS}}}{m_t^{\overline{\mathrm {DR}}}} \left( A_t- \frac{\mu }{\tan \beta }\right) + \frac{\mu }{\tan \beta }. \end{aligned}$$

(Except for $A_t$, which actually appears in the Feynman rules, FeynHiggs only pretends to perform these shifts but computes the sfermion masses using $m_t^{\mathrm {OS}}$.)

This procedure is available in FeynHiggs from version 2.11.1 on and is activated by setting the new value 2 for the runningMT flag. The comparison of the results with $\overline{\mathrm {DR}}$ and with OS renormalization admits an improved estimate of (some) of the missing three-loop corrections in the top/stop sector.

**Fig. 8:**
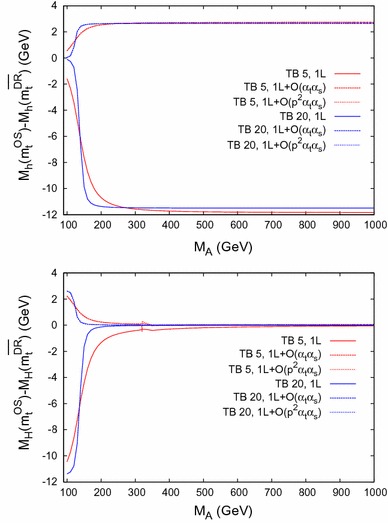
$\bar{\Delta }M_\phi = M_\phi (m_t^{\mathrm {OS}}) - M_\phi (m_t^{\overline{\mathrm {DR}}})$ for $\phi = h$ (*upper plot*) and $\phi = H$ (*lower plot*). The difference is shown as *solid* (*dashed/dotted*) *line* at the one-loop ($\mathcal{O}(\alpha _t\alpha _s)$/$\mathcal{O}(p^2\alpha _t\alpha _s)$) level as a function of $M_A$ for $\tan \beta = 5 (20)$ in *red* (*blue*) within Scenario 1

**Fig. 9:**
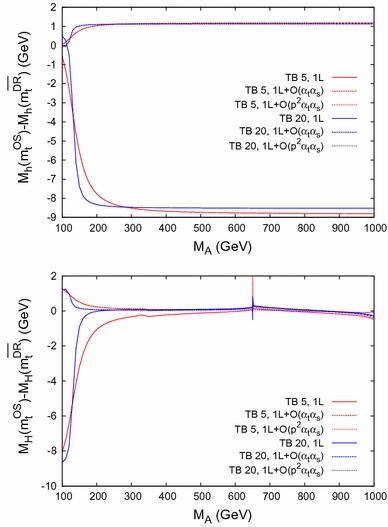
$\bar{\Delta }M_\phi = M_\phi (m_t^{\mathrm {OS}}) - M_\phi (m_t^{\overline{\mathrm {DR}}})$ for $\phi = h$ (*upper plot*) and $\phi = H$ (*lower plot*) as a function of $M_A$ within Scenario 2, with the same *line/color* coding as in Fig. 8. The peak in the *lower plot* originates from a threshold at $2\,m_{\tilde{t}_1}$. The threshold at $2\,m_t$ is suppressed by $1/\tan ^2\beta$

### Numerical analysis

In the following plots we show the difference

51 $$\begin{aligned} \bar{\Delta }M_\phi := M_\phi (m_t^{\mathrm {OS}}) - M_\phi (m_t^{\overline{\mathrm {DR}}}),\quad \phi = h, H, \end{aligned}$$

between $M_\phi$ evaluated in the OS scheme, i.e. using $m_t^{\mathrm {OS}}$ (*not*$m_t^{\mathrm {FIN}}$), and in the $\overline{\mathrm {DR}}$ scheme, i.e. using $m_t^{\overline{\mathrm {DR}}}$.

### Dependence on $\varvec{M_A}$

In the upper half of Fig. 8, $\bar{\Delta }M_h$ is plotted in Scenario 1 as a function of $M_A$ with $\tan \beta = 5 (20)$ in red (blue). The solid (dashed) lines show the difference evaluated at the full one-loop level (including the $\mathcal{O}(\alpha _t\alpha _s)$ corrections). The dotted lines include the newly calculated $\mathcal{O}(p^2 \alpha _t\alpha _s)$ corrections. For $M_A\gtrsim 200 \,\, \mathrm {GeV}$ one observes large differences of $\mathcal{O}(10 \,\, \mathrm {GeV})$ at the one-loop level, indicating the size of missing higher-order corrections from the top/stop sector beyond one loop. This difference is strongly reduced at the two-loop level, to about ${\sim } 3 \,\, \mathrm {GeV}$, now corresponding to missing higher orders beyond two loops from the top/stop sector. The dotted lines are barely visible below the dashed lines, indicating the relatively small effect of the $\mathcal{O}(p^2 \alpha _t\alpha _s)$ corrections as derived in Ref. [1].

**Fig. 10:**
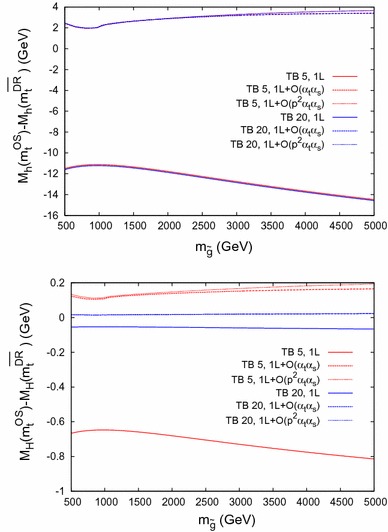
$\bar{\Delta }M_\phi = M_\phi (m_t^{\mathrm {OS}}) - M_\phi (m_t^{\overline{\mathrm {DR}}})$ for $\phi = h$ (*upper plot*) and $\phi = H$ (*lower plot*) as a function of $m_{\tilde{g}}$ within Scenario 1, for $M_A= 250 \,\, \mathrm {GeV}$ and with the same *line/color* coding as in Fig. 8

**Fig. 11:**
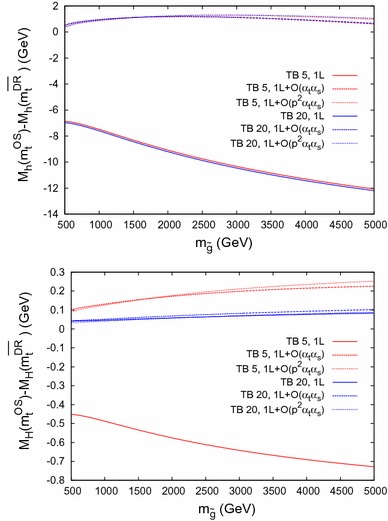
$\bar{\Delta }M_\phi = M_\phi (m_t^{\mathrm {OS}}) - M_\phi (m_t^{\overline{\mathrm {DR}}})$ for $\phi = h$ (*upper plot*) and $\phi = H$ (*lower plot*) as a function of $m_{\tilde{g}}$ within Scenario 2, for $M_A= 250 \,\, \mathrm {GeV}$ and with the same *line/color* coding as in Fig. 8

The lower plot of Fig. 8 shows the corresponding results for $\bar{\Delta }M_H$ with the same color/line coding. Here large effects are only visible for low $M_A$, where the higher-order corrections to $M_H$ are sizable (and the light Higgs boson receives only very small higher-order corrections). In this part of the parameter space the same reduction of $\bar{\Delta }M_H$ going from one loop to two loops can be observed.

The behavior is similar for Scenario 2, shown in Fig. 9 (with the same line/color coding as in Fig. 8), only the size of the difference $\bar{\Delta }M_h$ is $\sim 20\,\%$ smaller at the one-loop level, and $\sim 50\,\%$ smaller at the two-loop level compared to Scenario 1. The same peak structure due to thresholds as in Fig. 7 is visible.

**Fig. 12:**
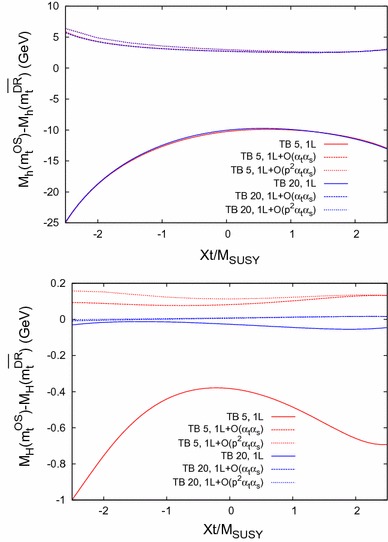
$\bar{\Delta }M_\phi = M_\phi (m_t^{\mathrm {OS}}) - M_\phi (m_t^{\overline{\mathrm {DR}}})$ for $\phi = h$ (*upper plot*) and $\phi = H$ (*lower plot*) as a function of $X_t= X_t^{\mathrm {OS}}$ within Scenario 1, with the same *line/color* coding as in Fig. 8

**Fig. 13:**
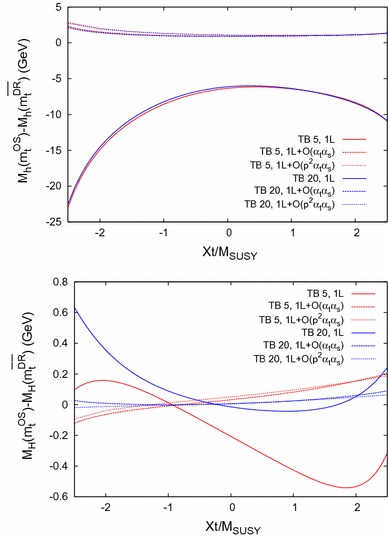
$\bar{\Delta }M_\phi = M_\phi (m_t^{\mathrm {OS}}) - M_\phi (m_t^{\overline{\mathrm {DR}}})$ for $\phi = h$ (*upper plot*) and $\phi = H$ (*lower plot*) as a function of $X_t= X_t^{\mathrm {OS}}$ within Scenario 2, with the same *line/color* coding as in Fig. 8

### Dependence on $\varvec{m_{\tilde{g}}}$

In Figs. 10 and 11 we analyze $\bar{\Delta }M_\phi$ as a function of $m_{\tilde{g}}$ in Scenario 1 and 2, respectively. We fix $M_A= 250 \,\, \mathrm {GeV}$ and use the same line/color coding as in Fig. 8. Due to the choice of an MSSM $\overline{\mathrm {DR}}$ top-quark mass definition, $m_t^{\overline{\mathrm {DR}}}$ varies with $m_{\tilde{g}}$ already at the one-loop level.

In the upper plots we show the light $\mathcal{CP}$-even Higgs-boson case, where it can be observed that the scheme dependence is strongly reduced at the two-loop level. It reaches 2–3$\,\, \mathrm {GeV}$ in Scenario 1 and $\sim 1 \,\, \mathrm {GeV}$ in Scenario 2, largely independently of $\tan \beta$. At the one-loop level the scheme dependence grows with $m_{\tilde{g}}$, whereas the dependence is much milder at the two-loop level. The effects of the $\mathcal{O}(p^2\alpha _t\alpha _s)$ corrections become visible at larger $m_{\tilde{g}}$, in agreement with Ref. [1].

The heavy $\mathcal{CP}$-even Higgs-boson case is shown in the lower plots. At small $\tan \beta$ scheme differences of $\mathcal{O}(600 \,\, \mathrm {MeV}(150 \,\, \mathrm {MeV}))$ can be observed at the one- (two-) loop level. For large $\tan \beta$ the differences always stay below $\mathcal{O}(50 \,\, \mathrm {MeV})$, in agreement with Fig. 8. The dependence on $m_{\tilde{g}}$ is similar to the light Higgs boson, but again somewhat weaker.

### Dependence on $\varvec{X_t}$

Finally, in Figs. 12 and 13 we analyze $\bar{\Delta }M_\phi$ as a function of $X_t= X_t^{\mathrm {OS}}$ in Scenario 1 and 2, respectively. We again fix $M_A= 250 \,\, \mathrm {GeV}$ and use the same line/color coding as in Fig. 8.

In the upper plots we show the light $\mathcal{CP}$-even Higgs-boson case. As before the scheme dependence is strongly reduced when going from the one-loop to the two-loop case. In general a smaller scheme dependence is found from small $X_t$, while it increases for larger $|X_t|$ values, in agreement with Ref. [93]. For most parts of the parameter space, when the two-loop corrections are included, it is found to be below ${\sim } 3 \,\, \mathrm {GeV}$. The contribution of $\mathcal{O}(p^2\alpha _t\alpha _s)$ remains small for all $X_t$ values.

In the heavy $\mathcal{CP}$-even Higgs-boson case, shown in the lower plots, the dependence of the size of the effects is slightly more involved, though the general picture of a strongly reduced scheme dependence can be observed here, too. In both scenarios, for large negative $X_t$ and $\tan \beta = 5$ the $\mathcal{O}(p^2 \alpha _t\alpha _s)$ contributions can become sizable with respect to the $\mathcal{O}(\alpha _t\alpha _s)$ corrections.

In conclusion, the scheme dependence is found to be reduced substantially when going from the pure one-loop calculation to the two-loop $\mathcal{O}(\alpha _t\alpha _s)$ corrections. This indicates that corrections at the three-loop level and beyond, stemming from the top/stop sector are expected at the order of the observed scheme dependence, i.e. at the level of ${\sim } 3 \,\, \mathrm {GeV}$. This is in agreement with existing calculations beyond two loops [54–56, 60].

A further reduction of the scheme dependence might be expected by adding the $\mathcal{O}(\alpha _t^2)$ contributions. The $m_t^{\overline{\mathrm {DR}}}$ value calculated at $\mathcal{O}(\alpha _s+ \alpha _t)$ is substantially closer to $m_t^{\mathrm {OS}}$, reducing already strongly the scheme dependence at the one-loop level. This extended analysis is beyond the scope of our paper, however.

## Conclusions

In this paper we analyzed the scheme dependence of the $\mathcal{O}(\alpha _t\alpha _s)$ corrections to the neutral $\mathcal{CP}$-even Higgs-boson masses in the MSSM. In a first step we investigated the differences in the $\mathcal{O}(p^2 \alpha _t\alpha _s)$ corrections as obtained in Refs. [1, 2]. We have shown that the difference can be attributed to different renormalizations of the top-quark mass. In both calculations an “on-shell” top-quark mass was employed. The evaluation in Ref. [1] includes the $\mathcal{O}(\varepsilon )$ terms of the top-quark mass counterterm, $\delta m_t^{\varepsilon }$, however, whereas this contribution was omitted in Ref. [2]. We have shown analytically that the terms involving $\delta m_t^{\varepsilon }$ do *not* cancel in the $\mathcal{O}(p^2\alpha _t\alpha _s)$ corrections to the renormalized Higgs-boson self-energies (an effect that was already observed in the $\mathcal{O}(\alpha _t\alpha _s)$ corrections in the NMSSM Higgs sector [90]). Numerical agreement between Refs. [1, 2] is found as soon as the $\delta m_t^{\varepsilon }$ terms are dropped from the calculation in Ref. [1]. Moreover, as an alternative interpretation, we have shown that omitting the $\delta m_t^{\varepsilon }$ terms is equivalent to a redefinition of the finite part of the two-loop field-renormalization constant which affects the Higgs-boson mass prediction at the three-loop order (apart from a numerically insignificant shift in $\tan \beta$ as an input parameter). The differences between the two calculations can thus be regarded as an indication of the size of the missing momentum-dependent corrections beyond the two-loop level, and they reach up to several hundred MeV in the case of the light $\mathcal{CP}$-even Higgs boson.

In a second step we performed a calculation of the $\mathcal{O}(\alpha _t\alpha _s)$ and $\mathcal{O}(p^2\alpha _t\alpha _s)$ corrections employing a $\overline{\mathrm {DR}}$ top-quark mass counterterm. We analyzed the numerical difference of the Higgs-boson masses evaluated with $\delta m_t^\mathrm{OS}$ and with $\delta m_t^{\overline{\mathrm {DR}}}$. By varying the $\mathcal{CP}$-odd Higgs-boson mass, $M_A$, the gluino mass, $m_{\tilde{g}}$ and the off-diagonal entry in the scalar-top mass matrix, $X_t$, we found that in all cases the scheme dependence, in particular of the light $\mathcal{CP}$-even Higgs-boson mass, is strongly reduced by going from the full one-loop result to the two-loop result including the $\mathcal{O}(\alpha _t\alpha _s)$ corrections. The further inclusion of the $\mathcal{O}(p^2\alpha _t\alpha _s)$ contributions had a numerically small effect. The differences found at the two-loop level indicate that corrections at the three-loop level and beyond, stemming from the top/stop sector, are expected at the level of ${\sim } 3 \,\, \mathrm {GeV}$. This is in agreement with existing calculations beyond two loops [54–56, 60]. The possibility to use $m_t^{\overline{\mathrm {DR}}}$ instead of $m_t^{\mathrm {OS}}$ has been added to the FeynHiggs package and allows an improved estimate of the size of missing corrections beyond the two-loop order.

## Footnotes

1 We concentrate here on the case with real parameters. For the case of complex parameters see Refs. [14–18] and references therein.

2 From a technical point of view we calculated the momentum-dependent two-loop self-energy diagrams numerically using the program SecDec [64–66].

3 Taking $\mathcal{O}(\varepsilon )$ terms into account in the expressions for on-shell counterterms beyond one loop is widely used in the literature; see e.g. Refs. [87–89].
